# Non structural protein of avian influenza A (H11N1) virus is a weaker suppressor of immune responses but capable of inducing apoptosis in host cells

**DOI:** 10.1186/1743-422X-9-149

**Published:** 2012-08-06

**Authors:** Sanjay Mukherjee, Shamik Majumdar, Veena C Vipat, Akhilesh C Mishra, Alok K Chakrabarti

**Affiliations:** 1 Microbial Containment Complex, National Institute of Virology, Sus Road, Pashan, Pune, 411021, India

## Abstract

**Background:**

The Non-Structural (NS1) protein of Influenza A viruses is an extensively studied multifunctional protein which is commonly considered as key viral component to fight against host immune responses. Even though there has been a lot of studies on the involvement of NS1 protein in host immune responses there are still ambiguities regarding its role in apoptosis in infected cells. Interactions of NS1 protein with host factors, role of NS1 protein in regulating cellular responses and apoptosis are quite complicated and further studies are still needed to understand it completely.

**Results:**

NS1 genes of influenza A/Chicken/India/WBNIV2653/2008 (H5N1) and A/Aquatic bird/India/NIV-17095/2007(H11N1) were cloned and expressed in human embryonic kidney (293T) cells. Microarray based approach to study the host cellular responses to NS1 protein of the two influenza A viruses of different pathogenicity showed significant differences in the host gene expression profile. NS1 protein of H5N1 resulted in suppression of IFN-β mediated innate immune responses, leading to down-regulation of the components of JAK-STAT pathway like STAT1 which further suppressed the expression of pro-inflammatory cytokines like CXCL10 and CCL5. The degree of suppression of host immune genes was found considerable with NS1 protein of H11N1 but was not as prominent as with H5N1-NS1. TUNEL assay analyses were found to be positive in both the NS1 transfected cells indicating both H5N1 as well as H11N1 NS1 proteins were able to induce apoptosis in transfected cells.

**Conclusions:**

We propose that NS1 protein of both H5N1 and H11N1 subtypes of influenza viruses are capable of influencing host immune responses and possess necessary functionality to support apoptosis in host cells. H11N1, a low pathogenic virus without any proven evidence to infect mammals, contains a highly potential NS1 gene which might contribute to greater virus virulence in different gene combinations.

## Introduction

The genome of influenza A viruses consists of eight segmented single stranded RNA with negative polarity which are capable of encoding a total of eleven known proteins [1]. The eighth and the smallest RNA segment encodes for the nonstructural protein NS1, which plays a significant role in overcoming host cellular defense mechanism and establishment of a productive infection [2]. It has been shown by many investigators that multifunctional NS1 protein is a major molecular determinant of virus virulence and contributes significantly in disease progression by modulating a number of virus and host-cellular processes [2-4]. The most widely studied function of the NS1 protein is to suppress host type I interferon (IFN-α/β) response which is one of the first innate immune response to virus infections. NS1 mediates this effect by two different mechanisms (i) NS1 directly interacts with RIG-1(Retinoic acid-inducible gene I) and PKR (Protein Kinase R) which play critical roles in detecting ssRNA and dsRNA respectively during Influenza A infection. Also, it inhibits pre-mRNA processing through interaction with CPSF30 (Cleavage and polyadenylation specificity factor). (ii) NS1 interact with host cellular mRNA and prevents its nuclear export [4-7].

Another important function of NS1 protein is to regulate host apoptotic mechanism. Apoptosis was initially thought to be a host cellular mechanism to restrict virus replication however, there are evidences now that it can be triggered by viral factors and can be used by the virus for its own benefit [8,9]. Both induction as well as suppression of apoptosis has been shown to be associated with NS1 protein [10-13]. Some studies have shown that NS1 protein specifically derived from H5 subtypes can induce apoptosis in human cells [10,14] however contrasting to that, other studies have shown suppression of apoptotic events by NS1 protein specifically derived from H1 subtypes in mammalian hosts [12,13]. Clearly, these observations were dependent on virus strain and cellular host system used for the study. The mode of NS1 expression in the host cells (i.e. through infection or transfection) also determined the apoptotic response [14,15]. Induction of apoptosis by NS1 protein was shown to be IFN- dependent in some cases through activation of NF-ĸB or IFN-independent through activation of caspases by different mechanism [13,14,16]. Inhibition of apoptosis, on the other hand was shown to happen through the activation of Phosphoinositide 3-kinase (PI3K) signaling pathway [15]. These observations clearly indicate that the role of Influenza A NS1 protein in host cells is very complex and needs further studies.

In this report, we compared the ability of NS1 proteins of two distinctly different subtypes of avian Influenza viruses (H5N1 and H11N1) to induce host cellular responses. Influenza A H5N1 belongs to highly pathogenic avian influenza viruses (HPAI) whereas, H11N1 is a low pathogenic atypical subtype of influenza viruses present in birds. In continuation of our earlier study of H11N1 viruses [17] we analyzed and found a great degree of sequence similarity in NS1 gene of H11N1 and HPAI- H5N1 influenza viruses. Using microarray based approach we studied host cellular gene expression response to NS1 protein of these two subtypes of influenza A viruses in order to have an insight into the role played by NS1 protein in modulating host cellular environment.

## Results

**Figure 1:**
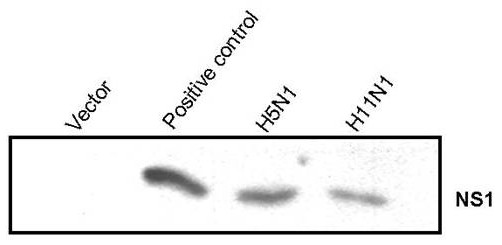
**Western blot analysis of NS1 protein expression in transfected cells.** Equal amount of cellular proteins isolated (10 μg) from transfected and control cell extracts were separated by 12.5% SDS-polyacrylamide gel electrophoresis. Proteins transferred to Hybond-C membrane were probed with specific monoclonal antibodies against Influenza A-NS1. A known Influenza A H1N1 NS1 construct was used as a positive control.

In order to understand whether NS1 protein from Influenza A viruses of widely varied pathogenicity have different ability to modulate host responses, a comparative analysis of host gene expression profile was carried out in transfected cells using microarray experiments. Expressions of NS1 protein from the control and transfected 293T cells were analyzed by western blotting. A 26 kDa band corresponding to the NS1 protein was observed in cells transfected with both H5N1 and H11N1 NS1 clones (Figure 1).

### Introduction of expression vector can induce host gene expression responses

**Figure 2:**
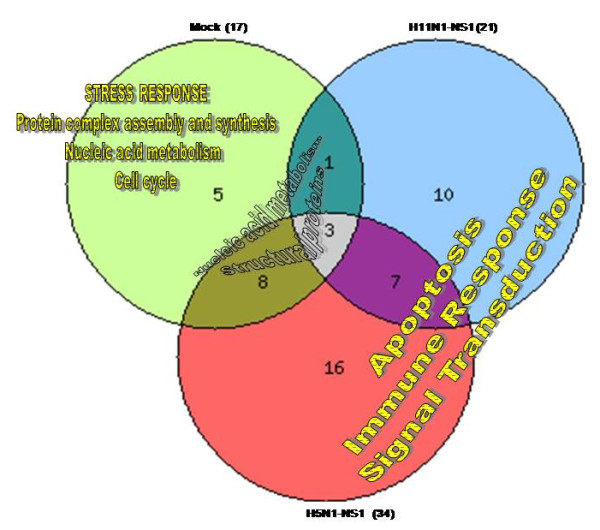
**A Venn-diagram representation of the number of host genes and the major pathways affected by NS1 protein of the two Influenza A viruses (H5N1 and H11N1) compared to mock.** The number of genes exclusively affected by vector (mock) are shown in light green (5) were found to be mainly involved in stress response. Genes exclusively affected by the transfection of NS1 protein are represented in light red (16) for H5N1-NS1 and light blue (10) for H11N1-NS1; Gene ontology analysis showed that these were mainly involved in apoptosis, immune response and signal transduction processes. The differentially expressed genes common between vector and H11N1-NS1 are shown in dark blue (1) whereas, genes common between vector and H5N1-NS1 are shown in dark green (8). Three genes were common in transfections with all the different plasmid constructs. All analyses are in comparison with control untransfected cells.

Interaction of expression vectors with cellular environment has been reported to result into a complex sequence of molecular and cellular responses that trigger innate and eventually adaptive immune responses [18,19]. In the present study microarray analysis resulted in identification of 17 host genes which were differentially expressing in response to transfection with the expression vector itself (Figure 2). These genes were mainly involved in stress response, protein complex synthesis and assembly, DNA metabolism and cell cycle. Genes involved in DNA replication and regulation of cell cycle like Eukaryotic translation initiation factor 4 gamma (EIF4G1), Ribosomal protein 19, Ribosomal protein 9, Small nucleolar RNA, Cyclin A2, CDKN1A, CDK2 and PCNA were found to be down-regulated by introduction of vector DNA inside the cells. Down-regulation of apoptotic factors like TNF-receptor was also observed in response to expression vector (Table 1A).

**Table 1:** A list of selected genes affected in the 293T cells by the transfection of A. expression vector [pcDNA3.0(-)] B. H5N1-NS1 clone and C. H11N1-NS1 clone

**A. Vector**		
**Gene ID**	**Gene Description**	**Fold change**
NM_000981	ribosomal protein L19; ribosomal protein L19 pseudogene 12	3.2
NM_002295	ribosomal protein SA pseudogene 9	2.6
NM_000994	small nucleolar RNA, H/ACA box 7A; small nucleolar RNA, H/ACA box 7B; ribosomal protein L32	3.1
NM_001728	basigin (Ok blood group)	2.7
NM_002086	growth factor receptor-bound protein 2	2.9
NM_001237	cyclin A2	-4.6
NM_000389	cyclin-dependent kinase inhibitor 1A (p21, Cip1)	-2.1
NM_002592	proliferating cell nuclear antigen	-2.6
NM_021138	TNF receptor-associated factor 2	-2.0
**B. H5N1-NS1**		
**Gene ID**	**Gene Description**	**Fold change**
NM_007294	breast cancer 1, early onset	-2.0
NM_000618	insulin-like growth factor 1 (somatomedin C)	-2.8
NM_000876	insulin-like growth factor 2 receptor	-2.1
NM_003327	tumor necrosis factor receptor superfamily, member 4	-2.1
NM_001904	catenin (cadherin-associated protein), beta 1, 88 kDa	-2.0
NM_003505	frizzled homolog 1 (Drosophila)	-2.2
NM_001527	histone deacetylase 2	-2.3
NM_004642	cyclin-dependent kinase 2 associated protein 1	-2.0
NM_004060	cyclin G1	2.0
NM_000075	cyclin-dependent kinase 4	1.5
NM_001067	topoisomerase (DNA) II alpha 170 kDa	2.2
NM_001924	growth arrest and DNA-damage-inducible, alpha	2.5
NM_002755	mitogen-activated protein kinase kinase 1	1.5
NM_002467	v-myc myelocytomatosis viral oncogene homolog (avian)	1.5
NM_001414	eukaryotic translation initiation factor 2B, subunit 1 alpha, 26 kDa	2.0
NM_004953	eukaryotic translation initiation factor 4 gamma, 1	2.0
NM_002156	heat shock 90 kDa protein 1 (chaperonin)	-2.3
NM_007315	signal transducer and activator of transcription 1, 91 kDa	-1.5
NM_002228	jun oncogene	-2.1
**C. H11N1-NS1**		
**Gene ID**	**Gene Description**	**Fold change**
NM_021138	TNF receptor-associated factor 2	-1.5
NM_001904	catenin (cadherin-associated protein), beta 1, 88 kDa	-1.5
NM_002228	jun oncogene	-1.5
NM_002750	mitogen-activated protein kinase 8	-2.0
NM_001924	growth arrest and DNA-damage-inducible, alpha	1.5
NM_002222	inositol 1,4,5-triphosphate receptor, type 1	2.0
NM_007315	signal transducer and activator of transcription 1, 91 kDa	1.5
NM_018955	ubiquitin B	3.2
NM_000459	TEK tyrosine kinase, endothelial	-2.1
NM_004428	ephrin-A1	-3.1
NM_006888	calmodulin 3 (phosphorylase kinase, delta)	2.3
X15183	heat shock protein 90 kDa alpha	-1.5

### Host gene expression responses to influenza A NS1: Comparison between NS1 protein of highly pathogenic H5N1 and low pathogenic H11N1 viruses

We examined global cellular gene expression levels in cells transfected with NS1 gene of two avian influenza viruses (H5N1 and H11N1) and compared them with untransfected control cells and the cells transfected with the vector DNA only. Cells which were transfected with H5N1-NS1 construct showed significant changes in expression of 34 genes whereas cells transfected with H11N1-NS1 construct showed differential expression of 21 genes (Figure 2). These 34 genes were mainly involved in immune response, regulation of gene expression, cell cycle, DNA replication and apoptosis. Out of 34 genes, 11 genes were found common with vector transfection and were mainly ribosomal proteins and cell cycle components. In spite of comparatively less number of host genes affected by H11N1-NS1, gene ontology analysis revealed that these differentially expressed genes were involved in similar biological pathways as with H5N1-NS1 transfected cells, indicating a generalized response to NS1 protein.

The genes which were exclusively affected by H5N1-NS1 included genes involved in DNA repair and nucleic acid metabolism like GADD-alpha, MAP2K1, Topoisomerase II and CDK4 (Table 1B). Up-regulated genes also included translation factors like eukaryotic translation initiation factor 2B, subunit 1 alpha and EIF4G1. However, genes MAPK8 and TNFR4 which are known to facilitate apoptotic mechanism were found to be down-regulated. HSP90 which is an anti-apoptotic gene was found to be down-regulated by both the NS1 proteins in the transfected cells (Table 1B and Table 1C). JUN protein, a known inducer of IFN signaling was down-regulated by NS1 protein of H5N1. Signal transducer and activator of transcription genes (STAT1, STAT2, STAT3 and STAT4) were observed to be up-regulated in H11N1-NS1 transfected cells as well as in case of mock transfected cells. However, these proteins were found to be down-regulated in H5N1-NS1 transfected cells indicting a greater ability of H5N1-NS1 protein to inhibit host innate immune response (Table 1B and Table 1C). IL8 and IL2 were also found to be down-regulated by H5N1 NS1 but not by H11N1-NS1.

**Figure 3:**
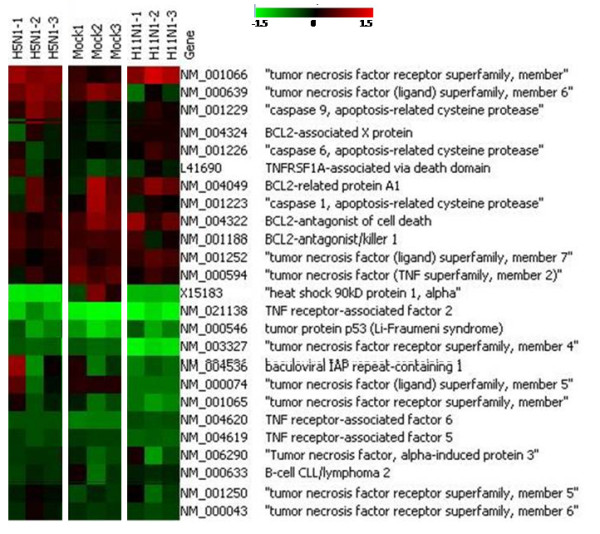
**Heat-map showing expression of apoptotic genes in 293 T cells transfected with pcDNA3.0 vector, H5N1-NS1 and H11N1-NS1 plasmid constructs.** The expression was compared with control (untransfected) cells. The maps are representative of 3 replicates of microarray experiment.

**Figure 4:**
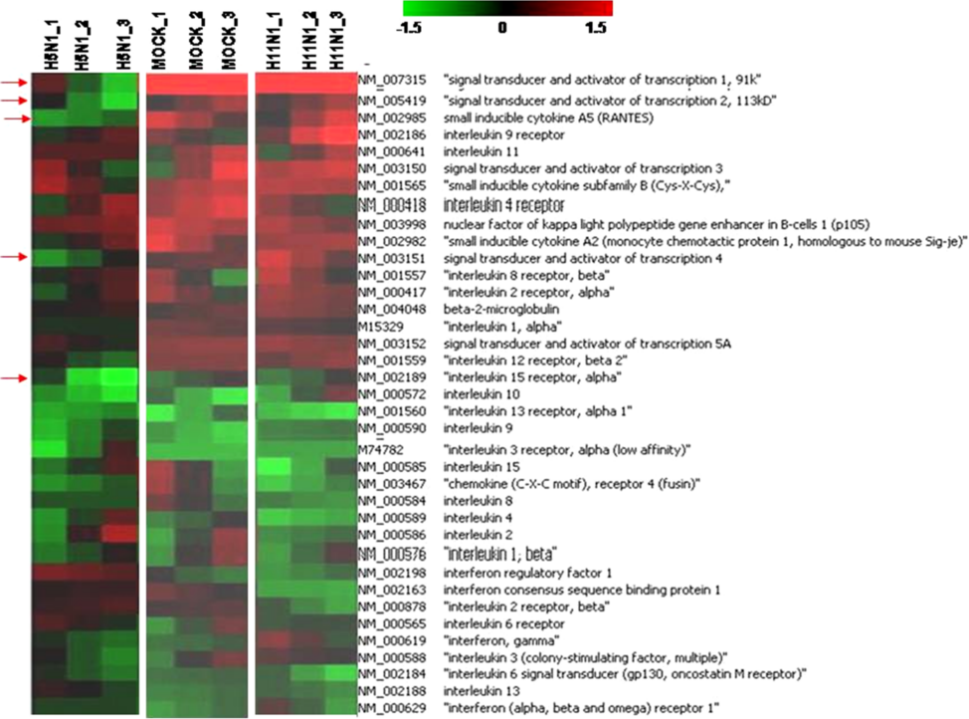
**Heat-map showing expression of immune genes in 293T cells transfected with pcDNA3.0 vector, H5N1-NS1 and H11N1-NS1 plasmid constructs.** The expression was compared with control (untransfected) cells. The maps are representative of 3 replicates of microarray experiment.

Immune response and apoptosis are the two major processes which are known to be modulated by influenza virus NS1 protein. Genes spotted on the microarray specifically involved in these processes were analyzed separately for the two NS1 transfection experiments using a different filtering criteria (+/- 1.5 folds for up/down regulation) (Figures 3 and 4). The overall expression profile of apoptotic genes in H5N1-NS1 transfected cells and H11N1-NS1 transfected cells was not different from mock transfected cells. Except for few genes like HSP90, TNF5 the expression trend was almost similar between mock and NS1 transfected cells. However, there was difference in the levels of expression of different apoptotic genes. For example there was higher degree of down-regulation of Tumor necrosis factor genes in NS1 transfected cells compared to mock cells (Figure 3). On the other hand, immune genes showed greater suppression in response to H5N1-NS1 transfection as compared to mock or H11N1-NS1 transfected cells. Genes involved in JAK-STAT pathway like STAT1 and inflammatory cytokines like CXCL10 and CCL5 (RANTES) were down-regulated in 293T cells transfected with H5N1-NS1 (Figure 4). As compared to H5N1-NS1, the expression profile of immune genes in H11N1-NS1 transfected cells was almost similar to mock cells apart from few genes like IL1-alpha and IL2 (Figure 4).

### Assessment of expression of selective host immune genes in response to NS1 protein by Real-time PCR

**Figure 5:**
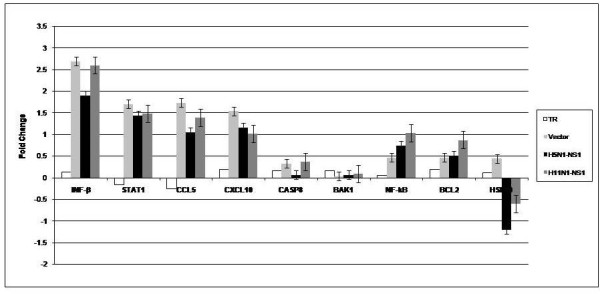
**Real-time PCR analysis of Immune and apoptotic genes in 293T cells.** Total RNA isolated from control transfected cells as well as from cells transfected with pcDNA 3.0 (-) vector, H5N1-NS1 construct and H11N1-NS1 construct were used for real-time PCR analysis. The expression was analyzed compared to controls (untrasfected cells). Expression of β-Actin gene was used as an internal control. The data is representative of 3 replicates. Error bars indicate mean+/- standard deviation.

Influenza A NS1 protein has been shown to cause immune suppression through IFN-alpha/beta signaling. To better understand the regulatory role of the NS1 protein, we used NS1 constructs of H5N1 and H11N1 influenza viruses and assessed transcriptional profiles of immune genes specifically involved in interferon mediated immune response in the transfected cells. The transcripts included were IFN-β, STAT1, CXCL10 and CCL5. We observed that relative to the H11N1, the H5N1 NS1 protein was able to suppress the expression of innate immune genes to a greater extend, specifically interferon *β* target genes. H5N1-NS1 protein showed a stronger capacity to inhibit the activation of IFN-*β* production as measured by IFN-*β* mRNA transcription. In contrast, H11N1- NS1 protein was found to be weaker in establishing an anti-IFN state and a poor suppressor of host immune genes. The differences in the abilities of the two NS1 proteins from different avian influenza A viruses to suppress the Interferon (IFN)-stimulated genes (ISGs) were clearly reflected by the mRNA expressions of IFN-β, STAT1 and pro-inflammatory cytokines like CXCL10 and CCL5 as shown by RT-PCR assays (Figure 5). We also studied expression of selective apoptotic genes like CASP8, BAK1 and HSP90 in the NS1 transfected cells. Apart from HSP90 gene none of the other genes showed significant expression difference compared to mock. HSP90 gene showed decrease expression in H5N1-NS1 transfected cells compared to control and mock cells as observed in microarray analysis but the decrease in expression of HSP90 in H11N1-NS1 transfected cells was not that significant.

### Expression of NS1 protein induces apoptosis in transfected cells

**Figure 6:**
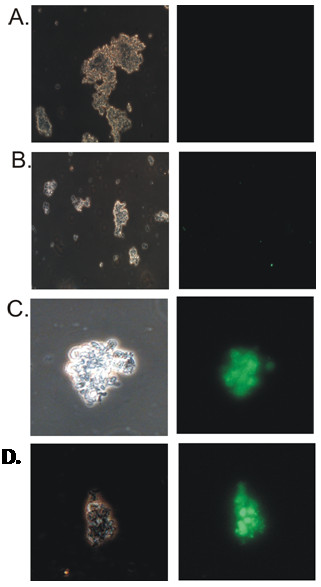
**TUNEL assay analysis of Apoptosis in control and NS1 transfected cells.** The cells were analyzed 24 h post transfection using fluorescently labeled anti-BrdU antibodies. The fluorescence was detected microscopically. Left panel are cells visualized under visible light without filter; Right panel are cells visualized under specific filters for fluorescence detection. The left and the right panel are the cells under same microscopic field **A**. Untransfected control cells **B**. Cells transfected with pcDNA 3.0 (-) vector **C**. cells transfected with pcDNA3-H5N1-NS1 construct **D**. cells transfected with pcDNA3-H11N1-NS1 construct.

Influenza A NS1 protein has been previously shown to cause apoptosis in cultured cells. To determine the apoptotic ability of the NS1 protein of the two avian influenza viruses we performed TUNEL assay in transfected 293T cells (Figure 6). Both H5N1 and H11N1-NS1 clones showed clear evidence to induce apoptosis in transfected 293T cells at 24 h post transfection. However, there was difference in the number of apoptotic cells in response to the two NS1 transfection. A comparatively higher number of cells were found to undergo apoptosis in response to H5N1-NS1 (40%) compared to H11N1-NS1 (20%) clearly indicating its greater apoptotic abilities (Table 2).

**Table 2:** Analysis of percentage of apoptotic cells in control, mock transfected and NS1 transfected cells as observed in TUNEL assay experiment

**Constructs**	**Percentage (%) of positive cells per field out of 10**^**6**^**cells used in TUNEL assay**
Control	0
Vector (pcDNA3.0)	0
H5N1-NS1	40%
H11N1-NS1	20%

The cells were analyzed 24 h post transfection using fluorescently labeled anti-BrdU antibodies. The fluorescence was detected microscopically. The percentage of fluorescent cells is represented as the degree of apoptosis in transfected cells.

### Protein sequences analysis

**Figure 7:**
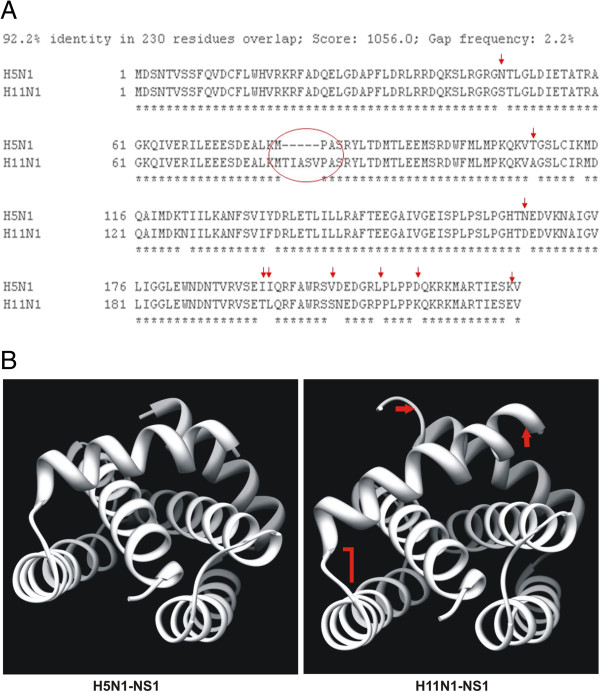
**A. Alignment of amino acid residues of NS1 protein of H5N1 and H11N1 influenza A viruses.** Red circle indicates the truncation region of H5N1-NS1 protein sequence; red arrows indicate the positions of amino acid mismatches between the two sequences. **B.** 3D Models of NS1 proteins from H5N1 and H11N1 viruses. The differences are highlighted in red arrows.

Alignment of the two NS1 protein sequences showed 12 amino acid variations between H5N1 and H11N1 influenza viruses. We also observed a 5 amino acid deletion in H5N1-NS1 sequence (Figure 7A). Three dimensional (3D) structural analysis of the NS1 protein of H5N1 and H11N1 viruses also revealed significant differences in protein folding as shown in Figure 7B. These differences in NS1 protein sequence and structure could have implications in the differences in host responses observed in our study.

## Discussion

Pathogenesis of influenza A viruses is a multigenic trait which involves the interaction of different viral proteins in host cells. NS1 protein of influenza A viruses works as a primary host defending factor by inhibiting IFN induction, which is considered as the most powerful innate defenses to limit viral replication [1,2,6]. Many reports are available to demonstrate how NS1 works in host cell to limit the production of IFN. Present study has clearly demonstrated that NS1 protein alone can influence the host innate immune response and is capable of inducing apoptosis in host cells which is largely dependent on viral subtypes. The ability of NS1 proteins derived from influenza viruses of widely diverse pathogenicity to suppress host IFN response supports the fundamental property of influenza NS1 protein in influencing host innate immune responses. We observed differences in the ability of the NS1 proteins of the two subtypes in suppressing host immune genes. This difference in the abilities of the two NS1 proteins can be explained on the basis of their protein sequences. Comparison of the NS1 amino acid sequences of the two viruses revealed 92% homology between them. Although belonging to a low pathogenic virus, H11N1-NS1 contains ESEV as the four terminal amino acids which play an important role in PDZ binding domain. This domain is a well known virulence marker for highly pathogenic avian influenza viruses [20]. However, differences in certain amino acids were also observed between the two NS1 sequences which could explain in part the differences in IFN mediated host responses. The ability to suppress IFN-*β* promoter activation was mapped to the C-terminal effector domain of NS1 protein while the RNA binding domain alone was unable to suppress IFN-*β* promoter inhibition [2]. A truncation of 5 residues at position 80-85 has been observed in NS1 protein of H5N1-virus towards the C-terminal region which could have occurred over time and added to the virulence of H5N1 influenza A viruses. Apart from this deletion there are 12 amino acid mismatches between the protein sequences (Figure 7A). Three dimensional (3D) structure analysis revealed that this sequence difference in the protein could actually result in structural differences between the NS1 proteins of the two influenza viruses and thus can have different interactions in the host cellular environment and therefore could result in different host responses as observed in our study (Figure 7B).

There are earlier reports which suggest that transfection of vector DNA itself can influence host cellular environment [18,19]. In our study we observed up regulation of stress, DNA metabolism, protein complex synthesis and assembly, and cell cycle modulating genes in response to vector DNA. However, when a functional NS1 protein expressed in 293T cells, it exerted different effects and influenced the cellular defense mechanisms which includes innate immune response and apoptosis. In the present study, analysis of microarray data revealed more number of host genes affected by influenza virus H5N1 NS1 protein as compared to H11N1 NS1. Research over last one decade on high pathogenicity and virulence of H5N1 influenza viruses have revealed many important aspects of the role of different influenza viral gene and protein including NS1 in viral pathogenicity. Although the multibasic amino acids stretch in the cleavage area between HA1 and HA2 of hemagglutinin is considered as the principle marker of virulence, other genes like PB2, NA, M also play important role in pathogenicity and spread of H5N1 infection [3]. In this study different NS1 proteins were expressed independently in 293T cells. NS1 proteins exerted differential host immune response indicating importance of the protein sequence in interactions with cellular host system.

Apart from defending immune response, the NS1 protein of influenza A viruses was shown to induce apoptosis in host cells [10,11,14], while other studies clearly indicated its role in inhibiting apoptosis [12,13]. It has been found from different studies that during virus infection (with wild type and/or NS mutated viruses) NS1 protein of influenza virus acts both as pro apoptotic and anti apoptotic manner depending on the viral subtype and host [13,14]. NS1 protein of highly pathogenic avian influenza A virus H5N1 could induce caspase-dependent apoptosis in human alveolar basal epithelial cells (A549), supporting its function as a proapoptotic factor during viral infection [14]. Recently it has been shown that heat shock protein 90 (HSP90) as a binding partner for the NS1 protein of both H5N1 and H3N2 strains and suggested that the NS1-HSP90 interaction might competitively promote the association of Apaf-1 with Cyt c and thus activate the caspase cascade [21]. In our study microarray analysis showed varied expression of apoptotic genes in NS1 transfected cells. Expression of TNF-R, CASP9, BIRC1 and BAK1 genes indicate activation of apoptotic mechanism in H5N1-NS1 transfected cells but expression of CASP1, CASP6, TNFRA5, TNFRA6 and p53 suggests anti-apoptotic role of NS1 protein. We also found down-regulation HSP90 in both H5N1-NS1 and H11N1-NS1 transfected cells which could play a role in inducing apoptosis as suggested by earlier reports. In order to understand the role of NS1 protein in apoptosis further, we performed TUNEL assay analysis. The assay revealed that both the viral derived NS1 proteins can induce apoptosis in the host cells. Since the gene expression analysis using microarray or real-time PCR could not give a clear picture of the expression of apoptotic genes, we believe that interaction of NS1 protein with apoptotic factors occur more at protein-protein level than at RNA level. The down-regulation of HSP90 is in accordance with earlier studies which could explain the activation of the cell death mechanism in transfected cells [21].

## Conclusion

Global gene analysis by microarray and its correlation with cellular functions clearly showed higher impact of NS1 protein of H5N1 origin as compared to H11N1. The high pathogenicity of H5N1 viruses can even be explained by the effect of a single protein of H5N1 virus in regulating cellular factors associated with viral virulence. Although H11N1 is a low pathogenic virus which has not been studied in detail, it has a potential NS1 gene capable of influencing host cellular environment even when expressed independently. NS1 of H11N1 might be an important virulence factor in a different viral genetic background.

## Materials and methods

### Cell line and viruses

Human embryonic kidney (293T) cells obtained from National Centre For Cell Science, Pune, India were grown in modified Eagle’s medium (DMEM; Invitrogen Life Technologies, Carlsbad, CA,USA) supplemented with 10% fetal bovine serum (FBS; Invitrogen Life Technologies),100 units/ml penicillin, 100 μg/ml streptomycin in tissue culture flasks (Corning, USA) at 37°C in a CO_2_ incubator. Influenza A/Chicken/India/WBNIV2653/2008(H5N1) [22] and A/Aquaticbird/India/NIV-17095/2007(H11N1) [17] viruses were grown and propagated in the allontoic cavities of 10-day old embryonated chicken eggs.

### Cloning of NS1 genes

Total RNA was isolated from influenza A H5N1 and H11N1 strains using viral RNA isolation kit (Qiagen, Germany) following manufacturer’s instructions. The RNA was reverse transcribed and the NS1 genes were amplified using subtype specific primers. Amplicons cloned into pcDNA 3.0(-) expression vector (Invitrogen, Carlsbad, CA) were screened by sequencing. Competent *E.Coli* DH5α cells were transformed with the plasmids. The plasmids containing NS1 inserts were isolated and purified using plasmid midi kit (Qiagen, Germany).

### Transient transfection and protein expression system

293T cells were transfected with pcDNA 3.0(-) control vector, pcDNA3.0-H5N1-NS1 and pcDNA3.0-H11N1-NS1 plasmid constructs using TransIT-LT1 (Mirus biosciences) transfection reagent according to the manufacturer’s protocol. Briefly, cells were cultured to monolayer in T-25 tissue culture flasks 24 hours before transfection. Plasmid DNA (6 μg/flask) and transfection reagent were mixed in serum-free medium and incubated for 30 minutes at room temperature following manufacturer’s protocol. Transfection complexes were then gently added into individual flasks. Cells were analyzed for gene and protein expression at 24 hours post transfection. Untransfected cells served as controls and cells transfected with the expression vector served as mock controls.

### Western blot analysis

Total cellular protein from control and transfected cells was isolated using RIPA lysis buffer [10X-20 mM Tris-HCl (pH 7.5), 150 mM NaCl, 1 mM EDTA,1 mM EGTA, 1% NP-40, Protease-Inhibitor cock-tail(Qualigens). Equal amount of proteins (10 μg) from cell extracts were separated by 12.5% SDS-polyacrylamide gel electrophoresis (12.5% SDS-PAGE) and transferred to Hybond-C (Amersham Biosciences) membrane with an electrotransfer apparatus (Cleaver Scientific Ltd) at 10 Volts (100 mA) for 1 h. The membrane was probed with specific monoclonal antibodies against Influenza A-NS1 protein (Santa Cruz Biotechnology, Inc. Santa Cruz, CA). Primary and secondary antibody interaction was performed in phosphate-buffered saline (pH 7.5). Proteins were visualized using the ECL detection system (Amersham Biosciences, USA).

### Microarray analysis

Total RNA was extracted from the control, mock transfected and NS1- transfected cells using Trizol reagent (Invitrogen Life Technologies, Carlsbad, CA, USA) and purified by the RNeasy kit (Qiagen, Germany) following standard methodology as described earlier [23,24]. Amplification of RNA and indirect labeling of Cy-dye was done by Amino Allyl MessageAmp II aRNA amplification kit (Ambion, Austin, TX, USA) using manufacturer’s instruction. One microgram of total cellular RNA from control and transfected cells was used for the experiments. The RNA was reverse transcribed and amplified. The purified amino allyl aRNA was labeled with Cy3 and Cy5 (Amersham Biosciences, USA) for control and experimental samples respectively. Purified samples were lyophilized, resuspended in hybridization buffer (Pronto Universal Hybridization kit, Corning, USA) and hybridized on the Discover human chip (Arrayit Corporation, Sunnyvale, CA). Hybridization was carried out in a Hybstation (Genomic Solutions, Ann Arbor, MI) and the conditions used were 55°C for 6 h, 50°C for 6 h, and 42°C for 6 h. Scanning was performed at 5-mm resolutions with the Scan array express (PerkinElmer, Waltham, MI). Grid alignment was done using gene annotation files and raw data were extracted into MS EXCEL.

Data was analyzed using GENOWIZ Microarray and Pathway analysis tool (Ocimum Biosolutions, Hyderabad, India). Data analysis was performed as described earlier [23]. In order to detect highly expressed genes, fold change analysis was done. Genes with 2 folds up/down-regulation were considered as differentially expressed at a p-value < 0.05, Student’s *t*-test. Functional classification of the genes was performed using gene ontology and pathway analysis. Microarray experiments were carried out in triplicates. The data is MIAME compliant and the raw data has been deposited in Gene Expression Omnibus (GEO) database No GSE39155.

### Quantitative RT-PCR using SYBR green I

The mRNA levels for IFN-β, STAT1, CCL5, CXCL10, CASP8, BAK1, HSP90 and BCL2 genes in control and transfected cells were analyzed by real-time RT-PCR. Total RNA was prepared from the control and transfected cells using RNeasy kit (Qiagen). One hundred nanograms (100 ng) of total RNA was used for quantitative RT-PCR analysis. Reaction was performed using the QuantiTect SYBR Green RT-PCR kit (Qiagen, Germany) according to the manufacturer’s instructions. Reactions were carried out on an ABI 7300 realtime PCR system (Applied Biosystems, Foster City, CA, USA) and the thermal profile used was Stage 1: 50°C for 30 min; Stage 2: 95°C for 15 min; Stage 3: 94°C for 15 sec, 55°C for 30 sec; and 72°C for 30 sec, repeated for 30 cycles. Melting curve analysis was performed to verify product specificity. Reactions were performed in triplicates. All quantitations (threshold cycle [CT] values) were normalized to that of β-Actin to generate ΔCT, and the difference between the ΔCT value of the sample and that of the reference was calculated as ΔΔCT. The relative level of gene expression was expressed as 2^-ΔΔCT^. Primer sequences for the genes of interest have been described earlier [23,24].

### Terminal deoxynucleotidyl transferase dUTP nick end labeling (TUNEL Assay)

The assay was carried out using TUNEL assay kit (Invitrogen) according to the instructions of the manufacturer. Briefly, Equal number of control, NS1-transfected and mock (vector) transfected cells(2 X 10^6^) were fixed in 1%(w/v) paraformaldehyde in 1XPBS(Phosphate buffer saline) for 15 minutes, washed in PBS and resuspended in 0.5 ml of PBS added with 70% Ethanol. The cells were kept at -20°C for 1hour. Labeling reactions were performed with BrdUTP using TdT enzyme for 60 min at 37°C in a humidified chamber. The labeled DNA was detected using Alexa Fluor 488 dye–labeled anti-BrdU antibody. Apoptosis was evaluated microscopically as flurescent cells per field at high-power magnification.

### Sequence analysis of NS1 proteins

H5N1 NS1 and H11N1 NS1 clones were directly used for cycle sequencing reactions. Sequencing was done on an automated Applied Biosystems’ 3130 XL system using cycle sequencing big dye terminator. The sequence of the NS1 genes cloned for the experiment are available in NCBI sequence database [Accession No CY055179 (H11N1 NS1) and CY046074 (H5N1 NS1)]. Alignment of H5N1 and H11N1 NS1 protein sequences was carried out using ClustalW program. The amino acid sequences were used to generate protein data bank (.pdb) files using SWISS-MODEL server (http://swissmodel.expasy.org/). The protein data bank (.pdb) files were used for visualization and generation of protein 3D structure using UCSF Chimera program (http://www.cgl.ucsf.edu/chimera).

## Competing interests

The authors declare that they have no competing interests.

## Authors’ contributions

AKC and ACM conceived the idea and initiated the project. AKC contributed to project design and supervised the project. SMU, SMA, AKC, VCV performed the experiments. AKC, SMU, SMA, VCV performed data analysis and bioinformatics studies. AKC, SMU and ACM wrote the paper. All authors read and approved the final manuscript.
